# Plasma Neutrophil Gelatinase-Associated Lipocalin as a Marker for Prediction of 3-Month Graft Survival after Kidney Transplantation

**Published:** 2017-02-01

**Authors:** A. Jafari, M. R. Khatami, S. Dashti-Khavidaki, M. Lessan-Pezeshki, A. Abdollahi

**Affiliations:** 1 *Assistant Professor of Clinical Pharmacy, Faculty of Pharmacy, Guilan University of Medical Sciences, Rasht, Iran*; 2 *Professor of Nephrology, Nephrology Research Center, Tehran University of Medical Sciences, Tehran, Iran*; 3 *Professor of Clinical Pharmacy, Nephrology Research Center, Tehran University of Medical Sciences, Tehran, Iran*; 4 *Professor of Nephrology, Nephrology Research Center, Tehran University of Medical Sciences, Tehran, Iran*; 5 *Associate Professor of Pathology, Vali-e-Asr Hospital, Tehran University of Medical Sciences, Tehran, Iran*

**Keywords:** Graft loss, Kidney transplantation, Neutrophil gelatinase-associated lipocalin

## Abstract

**Background:**

Ischemic injury during organ transplantation increases the risk of acute and chronic rejections by promoting alloimmune responses. Measurement of neutrophil gelatinase-associated lipocalin (NGAL) immediately after kidney transplantation may be promising for early detection of ischemic injuries to allograft.

**Objective:**

This study assessed possible predictive values of plasma NGAL levels during first hours after kidney transplantation for graft loss within the first 3 months after transplantation.

**Methods:**

45 kidney transplant recipients were classified into those without graft loss or with graft loss during 3 months after transplantation. Plasma NGAL levels were measured before and at 2, 6, 12, 24 and 96 hours after transplantation. Serum creatinine concentration was assessed daily during hospitalization and at 1, 2, and 3 months post-transplantation.

**Results:**

Serum creatinine and plasma NGAL levels were consistently higher in patients with graft loss compared with those without graft loss. At 2, 24, and 96 hours after transplantation, plasma NGAL concentration was significantly higher in patients who developed allograft loss within 3 months post-transplantation. The cutoff point of plasma NGAL at 2, 24, and 96 hours after transplantation for prediction of graft loss was 304.5 ng/mL (sensitivity of 71.4%, and specificity of 73.7%), 207.8 ng/mL(sensitivity of 85.7%, and specificity of 60.5%), and 184 ng/mL (sensitivity of 85.7%, and specificity of 71.1%), respectively.

**Conclusion:**

Plasma NGAL levels at 2, 24, and 96 hours after transplantation can predict 3-month graft loss with fair sensitivity and specificity.

## INTRODUCTION

Kidney transplantation is the treatment of choice in patients with end-stage renal disease. Kidney transplant recipients have better quality of life than patients undergoing dialysis [1]. Several factors such as recipient’s age, sex, and race; primary cause of renal failure; cold ischemia time; human leukocyte antigen (HLA) matching between kidney transplant recipient and donor; prior transfusion; and graft preservation method affect graft survival [2-4]. Acute rejection is a common complication after kidney transplantation and is associated with reduced graft survival [5]. Ischemic injury during organ transplantation is one the common early complications that increases the risk of acute and chronic rejections. Ischemic injury during transplantation potentiates the risk of acute rejection by promoting alloimmune responses and subsequently leads to long-term allograft dysfunction [6]. Even subtle ischemia-reperfusion injury is likely to have a negative impact on graft outcome. There are studies that suggest allograft biopsy at the time of engraftment (0-hour) because of its usefulness in detecting subclinical pathologic changes including subtle inflammation and immune activation [7, 8].

Therefore, we need to use non-invasive and sensitive markers to diagnose subclinical ischemic graft injury early and treat patients timely and individually to prevent future acute rejections and graft loss. Measurement of urine or blood biomarkers such as neutrophil gelatinase-associated lipocalin (NGAL), cystatin C, interleukin 18 (IL-18), and kidney injury molecule 1 (KIM)-1 immediately after transplantation for early prediction of acute kidney injury (AKI) have been proposed as the most promising biomarkers [9]. NGAL is a small 25 kDa molecule that belongs to the superfamily of lipocalins and is expressed at low levels in various epithelial cells, such as those in renal tubules and found in activated neutrophils [10]. NGAL is freely filtered by the glomerulus. It is largely reabsorbed in the proximal tubules by megalin-dependent endocytosis [11]. Thus, any urinary excretion of NGAL is likely only when there is concomitant proximal renal tubular injury that precludes NGAL reabsorption and/or increases *de novo *NGAL synthesis [11]. Although many studies have proposed urine [12-14] or blood [15-20] NGAL levels as one of the novel predictive biomarkers for early detection of delayed graft function after transplantation, its utility in prediction of long-term graft outcome has rarely been investigated. Conflicting findings have been published from the studies tested the prognostic value of urine [14, 21-24] or serum [24, 25] NGAL on graft function at longer time after transplantation.

In this study, we aimed at measuring serial plasma NGAL during the first hours after transplantation in recipients of deceased donor kidney and examining whether kidney injuries detected only by increased level of plasma NGAL can affect graft outcome within the first three months after transplantation regardless of early graft function. We also assessed predictive value of plasma NGAL during early hours after transplantation on graft function and survival within three months after transplantation.

## MATERIALS AND METHODS

**Study Design and Patients**

This cross-sectional study was conducted at kidney transplantation ward of Imam-Khomeini Hospital complex affiliated to Tehran University of Medical Sciences, Tehran, Iran. Forty-five patients aged more than 14 years who underwent kidney transplantation from deceased donor for the first time were included. All enrolled patients received the same immunosuppressive regimen according to our protocol. All patients received thymoglobulin induction and prednisolone, mycophenolate mofetil and tacrolimus as maintenance immunosuppressive regimen. Prednisolone dose was tapered down to 5 mg/day within one month after transplantation in all patients. The desired whole blood trough tacrolimus concentration was 8–12 ng/mL. All patients received intravenous ganciclovir/oral valganciclovir, oral cotrimoxazole and clotrimazole for prophylaxis of cytomegalovirus, *Pneumocystis jeroveci* pneumonia and candidiasis, respectively. The cold and warm ischemia times were kept shorter than one hour in order to minimize the impact of ischemia on the allograft. 

**Data Collection, Graft Function and Survival Assessment**

All demographic data (age, sex, weight, height) and considerable information before transplantation (serum creatinine, cause of renal failure or brain death, comorbid conditions) from the donor and recipient were gathered. Daily measurement of urine output and serum creatinine were started on the day of transplantation and continued until discharge of patient from the hospital. All patients were monitored within three months of transplantation; all aspects of graft function, serum creatinine, infections, and other complications were recorded. 

Creatinine reduction rate (%) was calculated as the difference between serum creatinine at the target day and the day before, divided by serum creatinine at the target day, multiplied by 100. Graft function three months after transplantation was evaluated by measuring serum creatinine level. In patients with graft loss, the last serum creatinine measured prior to dialysis restart, was considered for 3-month serum creatinine.

Graft survival was evaluated based on the number of patients’ grafts that were not lost within three months after kidney transplantation. Graft loss was considered if a patient needed maintenance dialysis within three months of transplantation. Graft losses due to vascular and urological surgery complications, ureteric obstruction, renal artery stenosis, and infections were not included. Number of possible or proven cellular/humoral rejection episodes within 3-month follow-up was also evaluated.

**Sample Collection and Plasma NGAL Assay**

A baseline blood sample was taken upon patient arrival to the transplant unit before transplantation. Post-transplantation samples were collected at 2, 6, 12, 24, and 96 hours following the transplantation. Samples were immediately processed and stored at 70 °C. We utilized a commercial double antibody sandwich enzyme-linked immunosorbent assay (ELISA) kit (Bioassay Technology Laboratory, Shanghai, China) for measuring plasma NGAL concentrations. Serum creatinine level was determined preoperatively, daily until hospital discharge, and at 1, 2, and 3 months after transplantation using Jaff´e method.

**Statistical Analysis**

Data were analyzed using SPSS^®^ for Windows^®^ ver19.0 (SPSS Inc., Chicago, IL, USA). Normality of variables was tested by the one-sample Kolmogorov-Smirnov test. Data are expressed as mean±SD for normally distributed variables and as median and IQR for variables not normally distributed. Baseline characteristics and biochemical data of the patients with and without graft loss were compared by two-sample *Student’s t* test. χ^2^ and Fisher’s exact tests were used in analyses of contingency tables. *Student’s t* test was used for samples with normal distribution; the Mann-Whitney U test was used for analysis of data not normally distributed. Bivariate correlations were analyzed using the Spearman’s *ρ* or λ correlation coefficient for non-parametric, and the Pearson’s correlation coefficient (r) for parametric measures of statistical dependence. A receiver operating characteristic (ROC) analysis was generated at 2, 6, 12, 24, and 48 hours after kidney transplantation to determine the cut-off value for plasma NGAL and serum creatinine and their potential to predict graft loss within 3-month follow-up. A p value of <0.05 was considered statistically significant.

Ethics

The study protocol was approved by the local Ethics Committee of Tehran University of Medical Sciences. Written informed consent was obtained from the recipients before enrolment.

## RESULTS

A total of 45 patients was assessed in this study. Demographic characteristics of the patients are shown in Table 1. Recipients with no residual urine output from their native kidneys showed no significant difference in the mean pre-transplant plasma NGAL (251.785±190.131 ng/mL) in comparison with that of recipients with residual urine output (338.582±417.63 ng/mL) in recipients with urine output of 50–1000 mL/24 hrs and 387.177±250.938 ng/mL in patients with urine output of >1000 mL/24 hours (p=0.322). Pre-transplant plasma NGAL concentration was not affected by the recipients’ age, sex, weight, body mass index, underlying kidney disease, mode or length of pre-transplantation dialysis, or pre-transplant plasma creatinine (Table 2). Six-hour urine output after kidney transplantation was correlated with 6-, 12-, 24-, and 96-hour plasma NGAL levels. The first 24-hour urine output was correlated with 24- and 96-hour plasma NGAL concentrations (Table 3). We found no significant correlation between plasma NGAL levels at all defined times and daily serum creatinine concentrations within the first week after transplantation (data not presented).

**Table 1:** Demographic and clinical characteristics of kidney transplant donors and recipients categorized by “graft loss” or “no graft loss” within three months of transplantation. Data are presented as mean±SD or n (%), as indicated

Characteristic	Total (n=45)	No graft loss (n=38)	Graft loss (n=7)	p value
Recipients				
Age (yrs)	41.4±14.6	42.5±13.7	35.6±20.1	0.413
Sex (Male) n (%)	28 (62)	23 (61)	5 (71)	0.693
BMI (kg/m^2^)	23.2±4.2	23.4±4.1	22.1±5.1	0.449
History of blood transfusion n (%)	24 (53)	21 (55)	3 (43)	0.689
Mode of dialysis n (%)				
Hemodialysis	40 (89)	33 (87)	7 (100)	0.577
Peritoneal dialysis	0 (0)	0 (0)	0 (0)	
No dialysis	5 (11)	5 (13)	0 (0)	
Time on dialysis (months)	28.9±18.4	29.6±18.9	25.4±16.9	0.59
Cause of kidney disease n (%)				
Hypertension	12 (27)	11 (29)	1 (14)	0.896
Diabetes mellitus	10 (22)	8 (21)	2 (29)	
Glomerulonephritis	3 (7)	3 (8)	0 (0)	
ADPKD	5 (11)	4 (11)	1 (14)	
Renal stone	2 (4)	2 (5)	0 (0)	
Bladder reflux	5 (11)	4 (11)	1 (14)	
Unknown	8 (18)	6 (16)	2 (29)	
Cumulative antithymocyte globulin dose (mg/kg)	6.7±2.1	6.5±2.0	7.3±2.8	0.387
Donors				
Age (yrs)	38.5±14.3	39.2±13.7	34.9±16.9	0.467
Sex (Male) n (%)	28 (62)	26 (68)	2 (29)	0.086
BMI (kg/m^2^)	25.8±4.0	25.9±3.9	25.5±5.0	0.799
SrCr (mg/dL)	1.3±0.3	1.3±0.3	1.1±0.3	0.302
SDC n (%)	39 (87)	34 (89)	5 (71)	0.230
Donor-recipient				
ABO complete match (%)	43 (96)	36 (95)	7 (100)	1
Sex match (%)	28 (62)	24 (63)	4 (57)	1

ADPKD: Autosomal dominant polycystic kidney disease; BMI: Body mass index; SCD: Standard criteria donor; SrCr: Serum creatinine concentration

**Table 2:** Correlations between the patients’ baseline plasma NGAL level and baseline recipients’ characteristics

Parameter	r	p value
Age (yrs)	-0.231	0.127
Gender	0.127	0.406
Weight (kg)	0.021	0.891
Body mass index (kg/m^2^)	0.128	0.400
Underlying kidney disease	-0.159	0.296
Mode of pretransplant dialysis	0.033	0.831
Length of pretransplant dialysis (months)	-0.098	0.548
Pre-transplantation serum creatinine (mg/dL)	0.04	0.796

**Table 3:** Correlations between the patients’ plasma NGAL levels and 6-hour and 24-hour urine output

Plasma NGAL, hours post-transplantation	6-hour urine output		24-hour urine output	
	r	p value	r	p value
2	-0.198	0.192	-0.181	0.233
6	-0.303	0.043	-0.253	0.094
12	-0.297	0.048	-0.243	0.107
24	-0.334	0.025	-0.321	0.032
96	-0.361	0.015	-0.301	0.044

Although plasma NGAL levels were higher in kidney transplant recipients with expanded criteria donor compared with those with standard criteria donors, the difference was not statistically significant. No significant correlation was also found between plasma NGAL levels at all times and recipients’ age or pretransplantation dialysis time (data not presented).

In addition, except for plasma NGAL level at 96 hours post-transplantation (r=0.335, p=0.025), plasma NGAL levels did not have any significant correlation with creatinine reduction ratios on post-transplantation day 2. It means that kidney transplant recipients with lower plasma NGAL measured 96 hours after transplantation had higher reductions rates in serum creatinine within two days of the transplantation. 

The median length of hospital stay after transplantation was 14 (IQR: 9.5–21) days. Plasma NGAL levels measured 2, 12, and 96 hours post-transplantation were strongly correlated with the length of hospital stay (r=0.3, p=0.045; r=0.323, p=0.031; and r=0.311, p=0.037, respectively).

Seven (16%) recipients experienced graft loss within three months of kidney transplantation. No significant difference was found between “graft loss” and “no graft loss” patients in terms of baseline demographic and clinical characteristics and cumulative thymoglobulin induction dose (Table 1). 

As expected, patients with graft loss within three months of transplantation had higher serum creatinine levels and lower creatinine reduction ratios during the first week post-transplantation, although these changes were not significant compared with “no graft loss” patients (Table 6).

**Figure 1:**
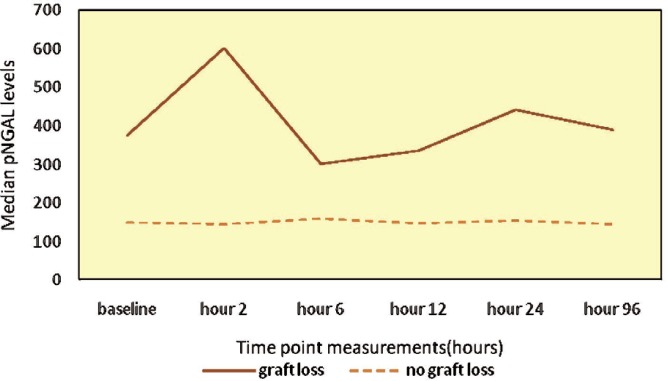
Evolution of plasma NGAL levels through the first hours post-transplant in patients with and without 3-month graft loss

Similar to serum creatinine, the median plasma NGAL concentrations in the first hours post-transplantation were consistently higher in patients with “graft loss” compared with “no graft loss” at most measured time points (Table 4 and Fig. 1). 

**Table 4:** Serial plasma NGAL levels through the first hours post-transplantation in patients with and without graft loss. Values are Mean±SD and Median (IQR

Plasma NGAL (ng/mL)	Before transplantation	Hours post-transplantation				
		2	6	12	24	96
No graft loss(n=38)	282.86±310.48 147.39 (104.46-336.75)	290.5±343.62 142.73 (106.75-325.37)	289.78±308.03 [156.11 (107.65-339.13)]	284.63±295.51 [145.613 (107.51-326.05)]	284.1±297.08 [151.61 (106.13-313.32)]	246.13±232.68 [142.34 (115.16-302.6)]
Graft loss(n=7)	521.53±465.9 375.7 (152.05–737.88)	596.76±418.16 602.04 (138.53–937.78)	589.76±656.68 300.36 (146.38–947.54)	635.98±699.66 333.75 (145.36–789.13)	525.61±333.34 441.51 (213.93–742.81)	518.69±424.15 390.06 (186.27–703.76)
p value	0.042	0.027	0.088	0.071	0.017	0.027

Compared with patients without graft loss, the changes in plasma NGAL over time in patients with graft loss were characterized by first increased in plasma NGAL concentrations from baseline values followed by a decrease in plasma NGAL levels and remaining high thereafter. 

**Figure 2:**
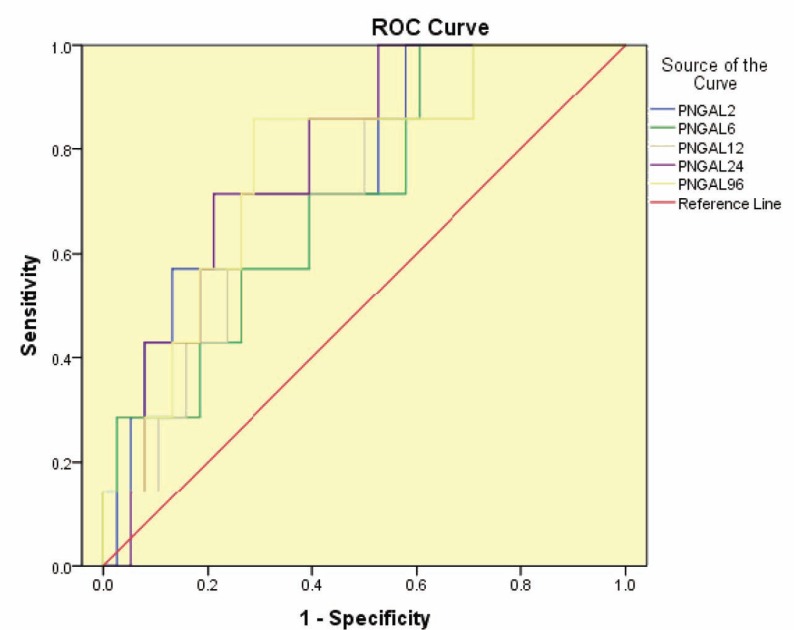
ROC analysis for plasma NGAL levels measured at 2, 6, 12, 24 hours post-transplantation to predict graft loss within three months of transplantation

ROC analysis showed that plasma NGAL levels in all time points could fairly an accurate predictor for graft loss within three months after transplantation (Fig. 2). Areas under the ROC curves (AUC), sensitivities, specificities, and the cutoff values for plasma NGAL levels are presented in Table 5. Serum creatinine concentrations at first seven days post-transplantation failed to predict graft loss based on ROC analysis (Table 6). 

**Table 5:** Area under the ROC curve, sensitivity, specificity, and cut-off point for plasma NGAL level during different hours post-transplantation for detection of graft loss within three months of the operation

Parameter	Cut-off point	Sensitivity (%)	Specificity (%)	AUC (95% CI)
Baseline NGAL	271.97	71.4	68.4	0.744 (0.585–0.904)
NGAL 2^nd^ hr	304.46	71.4	73.7	0.763 (0.583–0.943)
NGAL 6^th^ hr	167.85	71.4	60.5	0.707 (0.514–0.900)
NGAL 12^th^ hr	145.04	85.7	50	0.718 (0.525–0.911)
NGAL 24^th^ hr	207.85	85.7	60.5	0.782 (0.626–0.938)
NGAL 96^th^ hr	184.0	85.7	71.1	0.763 (0.580–0.946)

**Table 6:** Area under the receiver-operating characteristic curve during first 7 days post-transplantation for serum creatinine for predicting graft loss during three months after transplantation

Time after transplant (day)	Daily serum creatinine (mg/dL) in patient without graft loss (n=38) Mean±SD	Daily serum creatinine (mg/dL) in patients with graft loss (n=7) Mean±SD	p value	AUC (95% CI)
1	6.1±2.2	5.6±2.7	0.625	0.472 (0.177–0.677)
2	4.4±2.4	4.6±2.1	0.877	0.524 (0.302–0.746)
3	3.6±2.4	4.3±2.0	0.467	0.598 (0.377–0.818)
4	3.2±2.2	4.1±2.2	0.32	0.63 (0.403–0.856)
5	2.8±2.2	4.2±2.1	0.128	0.679 (0.461–0.897)
6	2.6±2.1	4.2±2.5	0.064	0.722 (0.498–0.946)
7	2.4±2.1	4.0±2.7	0.082	0.709 (0.47–0.948)

To evaluate the prognostic value of plasma NGAL levels for three-month graft function, we explored the correlation between plasma NGAL levels before transplantation and at the first hours post-transplantation with serum creatinine and estimated glomerular filtration rate (eGFR) assessed 1, 2, and 3 months post-transplantation. Although plasma NGAL levels showed positive correlation with serum creatinine concentrations and negative correlation with eGFR at the day of hospital discharge and also at 1, 2, and 3 months after transplantation in all patients, the correlations were not statistically significant (data not shown). ROC analyses were performed to assess plasma NGAL’s ability to predict poor graft outcome in the first, second, and third month after transplantation. Plasma NGAL levels in all time points were not significantly accurate for predicting low graft function (eGFR<60 mL/min/1.73 m^2^) 1, 2, and 3 months after transplantation.

To evaluate the prognostic value of plasma NGAL levels for 3-month rejection episodes number, we explored the correlation between plasma NGAL levels before transplantation and at the first hours after transplantation with total number of rejection episodes during first three months after transplantation. We found a significant correlation between plasma NGAL levels before (r=0.414, p=0.005) and 2 hours (r=0.425, p=0.004), 6 hours (r=0.387, p=0.009), 12 hours (r=0.444, p=0.002), 24 hours (r=0.352, p=0.018), and 96 hours (r=0.487, p=0.001) after transplantation with total number of rejection episodes during the first three months post-transplantation.

The episodes of polyomavirus, cytomegalovirus, and bacterial infections did not differ between patients with and without graft loss within three months after transplantation (Table 7). The episodes of acute cellular and humoral rejections within three months after transplantation in both groups are shown in Table 7. Two patients in graft loss group and three patients in no graft loss group experienced two episodes of acute cellular rejection within three months of transplantation. Cellular and humoral rejection episodes occurred more in patients with graft loss compared with patients without graft loss (Table 7). Correlation analysis showed that there was no association between number of cellular rejection episodes within three month after transplantation and occurrence of graft loss (λ=0). In contrast, strong and moderate association was observed between graft loss and number of humoral rejection episodes (λ=0.571) and total rejection episodes (λ=0.286).

**Table 7:** Total episodes of infections, acute cellular and humoral rejections within three months of transplantation

Parameter	“Graft loss” group	“No graft loss” group	p value
Number of polyoma virus infection episodes	0	1	0.664
Number of cytomegalovirus infection episodes	0	2	0.535
Number of bacterial infection episodes	3	17	0.844
Number of cellular rejection episodes	7	12	0.014
Number of humoral rejection episodes	6	2	<0.001
Total rejection episodes	13	14	<0.001

## DISCUSSION

This prospective study showed that plasma NGAL level is a promising biomarker for predicting allograft loss within three months after kidney transplantation. We assessed plasma NGAL levels during the first hours of day 1 to day 4 after transplantation in a cohort of 45 kidney transplant recipients. At 2, 24, and 96 hours after transplantation, plasma NGAL concentrations were significantly higher in patients who developed allograft loss within three months post-transplantation. Plasma NGAL level shortly after transplantation was higher in patients who were going to develop graft loss within three months of transplantation. In addition, changes in plasma NGAL concentrations over time showed different kinetic patterns between patients with and without 3-month graft loss (Fig. 1). Recipients who developed graft loss had higher levels of baseline plasma NGAL that rose further on the following hours post-transplantation, differing from patients without graft loss. 

We think early post-transplantation plasma NGAL level might represent graft injury and thus, might predict graft loss during following days to months. Using ROC analysis, findings suggested plasma NGAL concentrations in early days after transplantation as a good diagnostic test to identify patients with graft loss within three months of transplantation. The AUC-ROC for plasma NGAL was fair accurate (AUC: 0.7–0.8) for graft loss prediction within three months after transplantation. Determination of the paired sensitivity and specificity for the cutoff value of plasma NGAL was performed based on the closest to the left upper corner of the unit square to predict the graft loss. Two hours after surgery, a cutoff plasma NAL level of 304.46 ng/mL had a sensitivity of 71.4% and specificity of 73.7% for identification of graft loss. At 24 hours after surgery, a plasma NGAL cutoff value of 207.8 ng/mL had a sensitivity of 85.7% and specificity of 60.5% for identification of the graft loss. On the fourth day post-transplantation, a plasma NGAL level higher than 184 ng/mL predicted graft loss with a sensitivity of 85.7% and specificity of 71.1%.

So far, there has been no study to evaluate diagnostic and predictive values of plasma or urine NGAL for graft loss within early months after kidney transplantation using ROC analysis. In a study with longer follow-up period on pediatric kidney transplant recipients, no association was found between the amount of serum/urine NGAL at 1, 3, and 7 days post-transplantation and serum creatinine concentration at the first year after transplantation [24]. The mean serum and urine NGAL levels measured at 1, 3, and 7 days post-transplantation were not different between patients with GFR ≤80 mL/min/1.73 m^2^ and those with GFR >80 mL/min/1.73 m^2^ [24]. We also did not observe any association between plasma NGAL levels at assessed time points with serum creatinine and eGFR 1, 2, and 3 months after kidney transplantation.

Choi, *et. al.*, found that higher urine NGAL level on day two post-transplantation was associated with a significantly lower 1-year allograft eGFR. In multivariate logistic regression analysis, urine NGAL concentration measured on day two post-transplantation was an independent predictor for long-term poor graft function (defined as eGFR <60 mL/min/1.73 m^2^) in 62 kidney transplant recipients [23]. They suggested that a urine NGAL/creatinine ratio cutoff value of ≥153 ng/mg had a sensitivity and specificity of 95% and 65%, respectively (AUC-ROC: 0.83) for predicting “poor 1-year outcome” [23]. Although not statistically significant, our results also showed that plasma NGAL levels were negatively correlated with eGFR and positively correlated with serum creatinine after transplantation. Some studies [21, 22] evaluated correlation of urine NGAL with graft function for a longer follow-up period and suggest that perioperative urine NGAL levels are associated with poor 1-year allograft function. They showed that urine NGAL levels measured in the first days after kidney transplantation are predictive of graft function independent of donor characteristics, acute rejection episodes, and re-hospitalizations throughout the first year post-transplantation [21]. In contrast, other researchers [14] did not find any correlation between urine NGAL and renal function at one year post-transplantation but showed that urine NGAL levels within the first two weeks after transplantation are only correlated with renal function up to three months after transplantation. Another study showed that urinary level of NGAL can accurately predict graft function recovery up to three months post-transplantation [13]. Our results could not confirm this correlation. Our findings were in agreement with results reported by Hall, *et. al. *[25]. In their study, serum NGAL level at two days post-transplantation was not associated with 3-month graft function.

Hollmen, *et. al.* [14], suggested that day one urine NGAL level does not correlate with length of hospital stay after kidney transplantation. In contrast, our study showed that plasma NGAL levels in some time points correlated with length of hospital stay. 

In contrast to other studies [14, 21-23], we measured NGAL in plasma instead of urine. Use of urine NGAL as indicator of injury of proximal tubular cells, has been mentioned to be more specific compared to serum NGAL, which can be produced by other organs and released into the circulation following transplantation [21]. However, use of urine NGAL in kidney transplant recipients can also be a drawback because of possible transient graft anuria, which may preclude the availability of urine and consequently lack of enough sample to measure NGAL level. The persistent urine production by the native kidneys and the usual fluctuations of hydration status in these patients can also induce potential changes in urinary biomarkers concentrations, which can be considered another problem with measuring urine NGAL [26]. Although the genesis and sources of plasma and urinary NGAL require further clarification, benefit of use of plasma is that there is no need to exclude patients without residual urine output. Since we did not use urine for measurement of this biomarker, we could not accurately compare NGAL concentrations with results of studies that have evaluated the predictive value of urinary NGAL for graft function in long-term follow-up after kidney transplantation. Ramirez-Sandoval, *et. al.*, has proposed the hypothesis that urine NGAL is a more useful prognostic factor for post-operative subclinical tubular ischemic injury because significant extrarenal NGAL secretion in the recovery phase after surgery may mask any increased kidney secretion of NGAL into circulating blood [27].

According to the moderate association between the number of total rejection episodes within the first three months after transplantation and graft loss and evidence of strong association of number of humoral rejection within the first three months post-transplantation with graft loss, it seems that early identification and appropriate interventions for acute rejections ensure decrease in graft loss in long term.

In conclusion, plasma NGAL levels at 2, 24, and 96 hours after kidney transplantation predicted 3-month graft loss with fair sensitivity and specificity. Nowadays, plasma/urine NGAL as an early biomarker for diagnosis of delayed graft function, is suggested in several studies [12-16, 19], but its use is still not routine in clinical diagnosis of delayed graft function. However, there is no study to evaluate the predictive value of NGAL in early time points after transplantation for long-term graft loss. Our study suggested plasma NGAL levels as an early biomarker for graft injury within three months post-transplantation. Larger studies are needed to prove the accuracy of our findings.

Some advantages of our study included prospective design of the study and measurement of plasma NGAL levels at multiple early time points after kidney transplantation that provided the information about the pattern of change in plasma NGAL levels over time. However, we did not find any correlation between plasma NGAL concentration and graft function within three months post-transplantation. Nevertheless, it seems that following graft function for longer time after transplantation would be favorable. 

Our study has several limitations including the small sample size and being conducted as a single-center study. We suggest protocol biopsy for all patients to confirm even subclinical ischemic injuries and also use of other potential kidney injury biomarkers. 

Possibly, the results of this study and similar future studies help to early predict graft function in long term and consequently will assist clinicians to care about immunosuppressive regimen for patients with predicted worse graft function. 
